# A four-day Western-style dietary intervention causes reductions in hippocampal-dependent learning and memory and interoceptive sensitivity

**DOI:** 10.1371/journal.pone.0172645

**Published:** 2017-02-23

**Authors:** Tuki Attuquayefio, Richard J. Stevenson, Megan J. Oaten, Heather M. Francis

**Affiliations:** 1 Department of Psychology, Macquarie University, Sydney, New South Wales, Australia; 2 School of Applied Psychology, Gold Coast, Griffiths University, Queensland, Australia; Peking University, CHINA

## Abstract

In animals, a Western style diet–high in saturated fat and added sugar–causes impairments in hippocampal-dependent learning and memory (HDLM) and perception of internal bodily state (interoception). In humans, while there is correlational support for a link between Western-style diet, HDLM, and interoception, there is as yet no causal data. Here, healthy individuals were randomly assigned to consume either a breakfast high in saturated fat and added sugar (Experimental condition) or a healthier breakfast (Control condition), over four consecutive days. Tests of HDLM, interoception and biological measures were administered before and after breakfast on the days one and four, and participants completed food diaries before and during the study. At the end of the study, the Experimental condition showed significant reductions in HDLM and reduced interoceptive sensitivity to hunger and fullness, relative to the Control condition. The Experimental condition also showed a markedly different blood glucose and triglyceride responses to their breakfast, relative to Controls, with larger changes in blood glucose across breakfast being associated with greater reductions in HDLM. The Experimental condition compensated for their energy-dense breakfast by reducing carbohydrate intake, while saturated fat intake remained consistently higher than Controls. This is the first experimental study in humans to demonstrate that a Western-style diet impacts HDLM following a relatively short exposure–just as in animals. The link between diet-induced HDLM changes and blood glucose suggests one pathway by which diet impacts HDLM in humans.

## Introduction

The hippocampus is a brain structure long considered to be important for learning and memory [1]. An extensive body of animal data now suggests that a Western-style diet, characterised by high intakes of saturated fats and added sugars (an HFS diet) causes rapid impairments to hippocampal dependent learning and memory [2–6]. Consistent with these findings, are the observations in humans that poorer hippocampal-dependent learning and memory is associated with greater consumption of a Western-style diet [7–10]. In this study, we test whether HFS diets *cause* similar impairments in hippocampal dependent learning and memory in healthy lean young people.

Animals show quite clear impairments in hippocampal-dependent learning and memory (HDLM) when they are fed a diet high in saturated fat [11–14], high in sucrose [15–17], or high in both saturated fat and sucrose [18–27]. Importantly, such diet-induced impairments appear to be specific to hippocampally-based tasks, since non-hippocampal measures remain unaffected. For example, an HFS diet impairs hippocampal-dependent place-recognition memory while object-recognition memory (non-hippocampal) remains stable [2]. Another important consideration is how quickly a shift in diet from healthy rat chow to an HFS diet can impact HDLM. Impairments in a HDLM task can be found after 3 to 5 days exposure [2,23].

An important question based upon these animal findings is whether something similar happens to the human hippocampus when it is exposed to an HFS diet. Correlational evidence in humans has provided some support for these animal data. Greater consumption of an HFS diet is associated with impairments in HDLM in children [28,29], adults [7–10] and the elderly [30,31], suggesting that an HFS diet impacts HDLM across the lifespan. The claim that such a diet impacts HDLM rests upon the ability of certain neuropsychological tests, such as the delayed recall of stories or word lists, to selectively measure hippocampal function. This is supported by the following findings: (1) hippocampal damage severely impairs performance on such tasks [32]; (2) reduced hippocampal activation during fMRI is associated with poorer verbal memory recall [33,34]; and (3) hippocampal volume best predicts performance on such tests [35,36]. In addition, as in animals, diet-related cognitive effects appear to be specific to tests sensitive to hippocampal function, as cognitive tests not related to hippocampal function (attention and working memory) are unimpaired by HFS consumption [9]. In sum, there is clear evidence for a relationship between HFS diet and poorer HDLM in humans.

While findings from animal studies indicate that HDLM worsens as a consequence of consuming an HFS diet, this directional causal link has not been tested in humans. However, current human data suggests *improvements* in memory and executive function can occur, although not consistently, following reductions in energy intake and fat [37]. Briefly, performance on HDLM improves following a shift to a Mediterranean diet [38–40] or to a diet low in saturated fats and refined sugars [41,42]. If we consider experiments that have *increased* components of a Western-style diet over days or weeks, very few studies are available. Increasing *total* fat leads to deficits in working memory, attention and processing speed in healthy men after 5 days [43] and reaction time and attention in male athletes after 7 days [44]. However, these studies are problematic for the following reasons. First, these studies used a cognitive battery [45] using tests not dependent on hippocampal function, so it is unclear if HDLM performance would have changed from these diets. Second, changes in cognition were only evident on reaction time tasks [43,44], which improve following fat ingestion [46]. Third, since only changes in total fat were reported, conclusions regarding the effect of fat type on cognition are not possible. Thus, there is currently no human experimental evidence that components of a Western-style diet impair HDLM.

Though the hippocampus is traditionally associated with learning and memory, it also appears to be important for ingestive control [5,6]. One such ingestive control concerns the ability to perceive internal states such as hunger and satiety (i.e., interoception). Hippocampal lesions can produce impairments in accurately sensing signals of hunger and satiety (interoception) in animals [47] and humans [48,49]. Given the human and animal data suggest that exposure to a Western-style diet impairs hippocampal function, this should in turn result in downstream effects on the control of ingestive behaviour. This has been demonstrated in animal research showing that an HFS diet impairs the ability to use interoceptive cues of hunger and fullness [50–52]. Likewise, in humans, habitual consumers of a Western-style diet show reduced sensitivity to signals of hunger and satiety [9] and thirst [8], and an impaired ability to use such interoceptive cues to modulate appetitive behaviour [7]. Whether an HFS diet causes reductions in sensitivity to signals of hunger and satiety has not as yet been established in human studies.

Various neurobiological mechanisms may mediate the effects of an HFS diet on the hippocampus. Animal data show that HFS diets lead to marked deficits in glucoregulation, insulin sensitivity and relatedly elevated blood triglycerides [51,53], persistent increases in inflammation [2,18,54–56] and reductions in brain-derived neurotrophic factor [26,52,57–59]. In humans, there are similar links between impairments in memory and the aforementioned neurobiological mechanisms (for reviews, see [5,60]). In particular, poorer insulin sensitivity is associated with impaired cognitive performance [61–64] and adults with type 2 diabetes are also impaired on tests of delayed verbal memory [64–66]. This suggests that one likely and plausible mechanism by which diet may affect the hippocampus in humans may involve glucoregulation.

The present study sought to investigate the impacts of briefly consuming an HFS diet over four days relative to one lower in saturated fat and added sugar, on hippocampal related functioning. More specifically, we wanted to determine if, in a sample of lean healthy young adults who generally consumed a diet of adequate nutritional quality (i.e., one not characterised by high levels of saturated fat and added sugar), a four-day shift to an HFS diet would lead to: (1) poorer HDLM performance but with no change in control non-hippocampal measures; (2) reduced sensitivity to hunger and fullness; (3) differences in biological markers (i.e., blood glucose and lipids) and; (4) changes to diet outside of the laboratory manipulation (i.e., compensation)–all relative to controls.

## Materials and methods

### Participants

Participants were students recruited from Macquarie University between February 2014 and May 2016. Power analysis indicated that approximately 100 individuals (50 per group) were required in order to have an 80% chance of rejecting the null hypothesis if changes in the primary outcome variable (HDLM) were of a moderate effect size (*d* = 0.5–0.6) with an α = 0.05. Inclusion criteria involved participants reporting in a pre-study screen: (1) a BMI less than 25kg/m^2^; (2) a diet-screener score ≤60, indicative of a diet relatively low in saturated fat and added sugar for this student population (more below); (3) fluency in English; (4) no food allergies and omnivorous; and (5) not currently dieting. A total of 885 individuals started the screening process (detailed below) of which 244 were deemed eligible, and of whom 145 consented to participate. From this, 127 participants commenced the experiment, 25 failed to complete it, leaving 102 cases for analysis.

### Design

This study was a between-subjects experimental design with randomised allocation to one of two groups—one exposed to four days of breakfasts high in saturated fat and added sugar (Experimental group) and the other given a breakfast of similar palatability and food types (i.e., toasted sandwich, etc.), but significantly lower in saturated fat and added sugar (Control group). Participants were randomised to a group by order of arrival to the experiment. The primary outcome variable was change in HDLM measured at the start and at the end of the experiment (alongside other behavioural and physiological variables). All participants were tested at Macquarie University between February 2014 and May 2016.

### Breakfast meals

The breakfasts presented to each group comprised a toasted sandwich and a chocolate milkshake and these had markedly different nutrient profiles (see **Table 1**). Each of these breakfasts was pilot tested to ensure equivalent palatability and flavour profile. Twelve healthy lean participants consumed a half-portion sample (~250g) of each breakfast in counterbalanced order. Participants completed a set of ratings of how much they liked the sample, as well as ratings of how sweet, sour, bitter, salty, fatty and healthy they thought each sample was on a 7-point category rating scale (anchors 1 = *Not at all* and 7 = *Very*). This testing revealed no significant differences between the two breakfasts in terms of palatability, flavour profile, or ratings of healthiness (*p*s > .10).

**Table 1:** Nutritional breakdown of the breakfast by group.

	Experimental group	Control group
Total mass (g)	431	471
Energy (kJ)	3658	2941
Total fat (%)	53	17
Saturated fats (%)	30	6
Protein (%)	11	51
Carbohydrates (%)	36	32
Sugar (%)	18	11

### Measures

#### Pre-study screening tests

Potential participants were screened using an online battery of questionnaires. This battery was composed of: (1) a demographic and medical history questionnaire; (2) a 26-item food frequency questionnaire (scores ranging from 26 to 130) to measure added sugar and saturated fat intake–the Dietary Fat and Sugar Questionnaire (DFS: [67]); (3) the 18-item Three-Factor Eating Questionnaire (TFEQ-R18; [68]) to assess eating attitudes (cognitive restraint, uncontrolled eating, and emotional eating); (4) the International Physical Activity Questionnaire-short form (IPAQ-SQ; [69]) to assess physical activity; and (5) the 10-item Kessler Psychological Distress Scale (K-10; [70]) to determine current mental well-being. Participants were excluded from the study if they reported a BMI > 25 kg/m^2^, a DFS score > 60, were currently dieting or reported any food allergies.

#### Hopkins Verbal Learning Task-Revised (HVLT)

The HVLT [71] is a 12-item word list that is read to the participant three times, requiring recall after each presentation. Following a 20–25 minute delay, participants are asked to recall all the words they remember from that list (delayed recall). This is followed by recognition test, which was not used here due to lack of variance. The HVLT-R has six alternate forms that make it ideal for repeat testing, and these were counterbalanced across participants.

#### Logical Memory (LM)

The LM test [72] involves listening to two short stories. Participants are then asked to repeat back as many details as possible immediately after hearing the story and then again after a 20–30 minute delay. The standard Wechsler Memory Scale (WMS) IV stories were used in combination with six alternate stories (counterbalanced across participants), whose structural and statistical properties were compatible with the standard WMS-III [73] and WMS-IV stories [74].

#### National Adult Reading Test (NART)

The NART [75] is 50-item single word reading test of graded difficulty, where all words are irregular and violate grapheme-phoneme correspondence rules (e.g., ache, thyme, topiary) and was used here as a measure of intelligence [76] to check for any group differences in this variable.

#### Digit Span (DS) test of the Wechsler Memory Scale-III (WMS-III)

Participants were read a sequence of numbers of increasing length (two trials per sequence), which they then had to repeat back either in the same order as delivered or in reverse order. This test, drawn from the WMS-III [77] was administered as per the manual, to determine any general decline in cognitive functioning or motivation across the experiment.

#### Biological measures

Height and weight for each participant were used to calculate body mass index (BMI). Waist circumference was also measured. Blood glucose and triglycerides measurements were taken using the CardioChek PA® Analyzer. Measurements ranges in each test were 20-600mg/dL for blood glucose and 50-500mg/dL for triglycerides. One 50μL whole blood sample was taken for both measurements using a fingerstick method (UniStick®3 21 Gauge 2.0mm depth).

#### Food diary

Participants were required to fill in an online food diary to track their daily eating behaviours (i.e., energy and macronutrient intake) prior to and during the experiment. Participants were asked to record, in as detailed a manner as possible, every item that they had eaten and drunk, the time they consumed it, the amount consumed and how it was prepared. Participants could estimate portion size using household measures, weight on packaging or from 15 sets of colour photographs depicting small, medium and large portions of frequently consumed foods [78]. The food diary was adapted from that developed by the Medical Research Council collaborative centre for Human Nutrition Research (Cambridge, UK). The food diary data were analysed using FoodWorks 8 Premium software, which uses food composition data from several sources including 5740 Australian foods and beverages [79] and 7906 food items from the United States [80].

#### Interoception and current mood ratings

Participants completed a set of ratings for how hungry, full, thirsty, alert and happy they were plus how strong their appetite for something sweet was, and how strong their appetite for something savoury was–in that order–each on a separate 7-point category rating scale (anchors *1 = Not at all* and *7 = Very*).

### Procedure

The study protocol was approved by the Macquarie University Human Research Ethics Committee and written consent (including notification of their right to withdraw without penalty at any time) was provided by each participant, with debriefing of the detailed study aims at the end of the experiment. In the week preceding the study, participants were asked to complete the food diary on two occasions to determine their pre-study dietary habits, from which a daily average was computed (noting that there were no significant differences in daily intakes on the two occasions). On the first experimental day (Day 1), and following an overnight fast (with no restrictions on fluid intake), participants arrived for testing between 6am and 12pm. They then attended the laboratory at the same time for the remaining three consecutive days of testing.

On Day 1, participants completed the immediate recall measures of the LM and HVLT tests, followed by the Digit Span and NART, and had their height, weight and waist circumference measured. A whole blood sample was then taken to measure blood glucose and triglycerides. Participants were then asked to recall the stories and words from the LM and HVLT tasks, respectively. The first set of interoception and mood ratings were then completed, and this was followed by the breakfast meal, with each participant asked to consume as much of it as possible. Participants were left alone for twenty minutes to eat and were given the option to watch TV or read a magazine. Following this, the experimenter removed all uneaten food for later weighing. Participants then completed a second set of interoception and mood ratings, post-prandial neuropsychological (LM, HVLT and DS) and blood tests (blood glucose and triglycerides) in the same manner and order as above (excluding the NART). That is, all measures (except the NART and BMI) were administered both before and after the breakfast meal. Participants were allowed to leave and requested to fill in the food diary for the remainder of Day 1.

Participants attended the lab on Days 2 and 3 to consume breakfast, completing the interoception and mood ratings prior to and following the breakfast. Again, each breakfast was weighed before and after consumption to assess food intake. While the breakfasts and interoception ratings were given across the four consecutive test days, neuropsychological and blood tests were only administered on Day 1 and 4 of the study. The procedure on Day 4 was identical to Day 1 (except the NART was not repeated). Participants were then requested to fill out the food diary for the remainder of Day 4 (noting that Day 1 and 4 diary entries did not differ and these were collapsed for analysis).

### Analysis

All neuropsychological measures were scored as per their respective manuals. For the memory measures, percent retention scores were computed for both the HVLT (delayed recall trial/ highest score taken from immediate recall trial 2 or 3) x 100) and for LM (delayed recall/ immediate recall) x 100). For Digit Span two scores were computed; forward recall of numbers (Digit Span forwards; score range 0–16) and the reverse recall of numbers (Digit Span backwards; score range 0–14). The HVLT data for Day 1, first test were non-normal, and this was driven by one outlying value. This data point was replaced by their less extreme Day 1 second test value. Coefficient alphas across the 4 testing occasions were adequate for HVLT retention (α = 0.70), poor for LM retention (α = 0.52), and good for Digit Span forwards (α = 0.89) and backwards (α = 0.88).

Three other variables were identified as being non-normal–the interoceptive data, blood triglycerides, and participants age, and these were all transformed (root for the former two, reciprocal for the latter) enabling parametric testing. For the biological measures, coefficient alphas were adequate for blood glucose (α = 0.62) and good for triglycerides (α = 0.88).

Multiple ratings of current mood and cravings for savoury and sweet foods were obtained during the study. Preliminary analyses revealed no significant effects of interest and so these ratings are not further reported. A two-tailed alpha of 0.05 was used for all reported tests, with all analyses being conducted using SPSS version 21.

## Results

### Participant characteristics

A total of 102 participants completed the study and their characteristics are summarised by Group in **Table 2**. The two groups were similar in age, mental well-being (K-10), exercise habits (IPAQ), eating attitudes (TFEQ) and estimated IQ (NART). Some differences were noted in BMI and waist circumference, which were significantly higher in the Control group, alongside a trend for greater habitual consumption of saturated fat and added sugar (DFS) in this group as well. While there was no significant group difference in Gender distribution, we note that there was nearly double the number of men in the Control group, relative to the Experimental group. To control for any effect of these differences–and recalling that participants were randomly assigned to Groups–these identified variables (BMI, waist circumference, DFS & gender) were included as covariates (following Z-transformation) in all of the analyses.

**Table 2:** Baseline descriptive statistics for each group.

	Descriptive statistics, Number/Mean (SD)		Difference
	Experimental group	Control group	p-value
Number	51	51	-
Gender (Female/Male)	44/7	38/13	0.14
Age	20.2 (3.9)	21.0 (6.6)	0.50
BMI	20.8 (2.3)	22.1 (2.5)	0.01*
Waist circumference (cm)	72.9 (7.3)	75.9 (6.3)	0.03*
DFS (diet) score	51.4 (6.7)	53.6 (5.7)	0.07
K-10	18.5 (6.3)	18.8 (5.2)	0.78
IPAQ	9.8 (3.2)	10.7 (3.8)	0.20
TFEQ-R18 (Cognitive restraint)	13.1 (3.5)	13.6 (3.3)	0.52
TFEQ-R18 (Uncontrolled eating)	15.5 (5.3)	16.5 (3.8)	0.25
TFEQ-R18 (Emotional eating)	5.8 (2.4)	6.2 (2.3)	0.45
NART Full Scale IQ	108.1 (5.8)	108.7 (5.1)	0.57

BMI: Body mass index; DFS: Dietary Fat and Sugar questionnaire; K-10: Kessler-10 Psychological distress scale; IPAQ: International Physical Activity Questionnaire; TFEQ-R18: Three Factor Eating Questionnaire- Revised; NART: National Adult Reading Test.

* *p* < .05

### Experimental manipulation

Participants eating the Experimental breakfast consumed more energy, total fat, saturated fat, total carbohydrates and sugar (*p*s < .001), but less protein (*p* < .001) at breakfast, than those eating the Control breakfast (see **Table 3**). As our principal interest was in the effect of dietary composition (i.e., added sugar and saturated fat) rather than total energy intake on the test breakfasts, and also because individuals within each group varied in how much of each breakfast they consumed–we used total energy intake (Z-transformed) at breakfast as a further covariate (except in analyses where energy intake was already included within the dependent variable–more below).

**Table 3:** Nutritional breakdown of breakfasts *consumed*, averaged across test days.

	Descriptive statistics, Mean (SD)		Difference
	Experimental group	Control group	p-value
Volume	423 (65)	435 (65)	.37
Energy (kJ)	3593 (550)	2716 (412)	.00*
Total Fat (%)	53.0 (0.7)	15.9 (2.1)	.00*
Saturated fat (%)	30.3 (0.1)	5.3 (0.6)	.00*
Total carbohydrates (%)	35.7 (1.4)	31.8 (0.6)	.00*
Sugars (%)	17.7 (2.5)	10.0 (0.6)	.00*
Protein (%)	11.5 (0.7)	51.3 (3.1)	.00*

Exp. = Experimental group

Sig* = significance level p < .05 of independent samples t-test

Sig^ = significance level p < .05 of repeated measures ANCOVA for Week by Group interaction; kJ = kilojoules.

### Neuropsychological measures

#### Hopkins-Verbal Learning Test (HVLT)

The HVLT percent retention data were analysed with a three-way mixed design ANCOVA, with Day (Day 1[pre-exposure] vs. Day 4 [post-exposure]) and Time (pre-breakfast vs. post-breakfast) as the within factors and Group (Control vs. Experimental) as the between factor, with BMI, waist circumference, average breakfast energy intake, DFS score, and gender as covariates.

**Fig 1:**
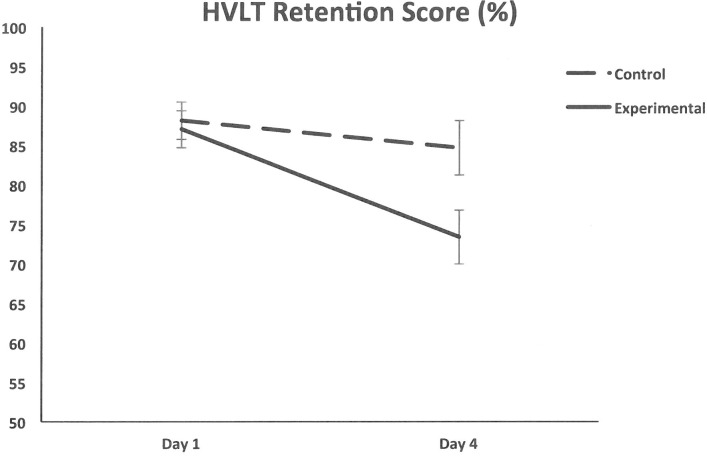
HVLT scores. Mean (± standard error) HVLT retention score (%) on test days one and four for the Experimental and Control groups.

The ANCOVA revealed a main effect of Day (*F*(1,94) = 24.54, partial eta-squared = 0.21, *p* < 0.001), which was qualified by an interaction between Day and Group (*F*(1,94) = 4.54, partial eta-squared = 0.05, *p* = 0.038). Further pairwise comparisons revealed that retention was significantly poorer in the Experimental group at the end of the study relative to the start (*M* = -15.7%, *p* < 0.001), but not in the Control group (*M* = -3.9%, *p* = 0.251; see **Fig 1**) consistent with our hypothesis. Moreover, if we consider *only* the HVLT retention scores before breakfast, the Day by Group interaction is still significant, (*F*(1,94) = 4.45, partial eta-squared = 0.045, *p* = 0.038), indicating that prandial-related effects did not interfere with our observation of diet-induced memory impairment.

Prandial-related effects were also observed, with a main effect of Time (*F*(1,94) = 47.46, partial eta-squared = 0.34, *p* < 0.001), which was qualified by an interaction between Time and Day (*F*(1,94) = 12.84, partial eta-squared = 0.12, *p* = 0.001). Retention was poorer after breakfast than before, with this drop being larger on Day 4 (*M* = -19.5%) than on Day 1 (*M* = -7.9%). There were no other significant effects.

#### Logical Memory (LM)

The LM percent retention score data were analysed using the same three-way mixed design ANCOVA described above. The only significant outcome was a main effect of Group (*F*(1,94) = 5.33, partial eta-squared = 0.05, *p* = 0.023), with the Experimental group (*M* = 85.9%) performing more poorly overall than the Control group (*M* = 91.9%).

#### Forward and backward digit span

The forward and backward digit span data were analysed separately in two further three-way mixed design ANCOVAs. For the forward digit span data, there were significant main effects of Day (*F*(1,94) = 15.57, partial eta-squared = 0.14, *p* < 0.001) and Time (*F*(1,94) = 4.46, partial eta-squared = 0.05, *p* = 0.037) and an interaction between these two variables (*F*(1,94) = 5.75, partial eta-squared = 0.06, *p* = 0.018). Forward digit span increased from Day 1 (*M* = 10.5) to Day 4 (*M* = 11.1), with a larger prandial improvement evident on Day 1 (*M* change = 0.5) relative to Day 4 (*M* change = 0.1). There were no other significant effects. For the backward digit span data, only one effect was significant, Day (*F*(1,94) = 20.59, partial eta-squared = 0.18, *p* < 0.001). Participants backward digit span increased from Day 1 (*M* = 6.9) to Day 4 (*M* = 7.5). As no alternate forms of either forward or backward digit span were used, these improvements may reflect the effects of practice.

### Interoceptive measures

**Fig 2:**
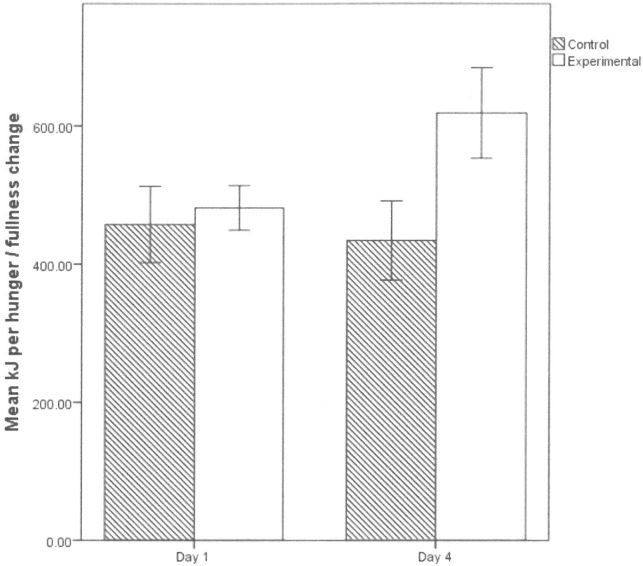
Interoception scores. Mean (± standard error) kilojoules (kJ) required to shift hunger and fullness ratings on days one and four for each group.

For Day 1 and Day 4 respectively, the energy consumed at Breakfast by each participant was divided by their change in hunger and fullness ratings combined (as these two variables significantly correlate, median r = -0.51), with the resulting group means displayed in **Fig 2**. This value (the interoception score) reflects the number of kilojoules (kJ) required to shift hunger and fullness ratings by 1 point. Following transformation, as the variables were non-normal, and with Day 4 interoception score serving as the dependent variable, we conducted a univariate ANCOVA, with Group as the between factor, and Day 1 interoception score, BMI, waist circumference, DFS score, and gender as covariates.

The ANCOVA revealed a significant main effect of Group (*F*(1,93) = 7.43, partial eta-squared = 0.07, *p* = 0.008). Thus, after controlling for differences on Day 1 interoception score, the Experimental group became significantly less sensitive to the effects of the breakfast, requiring more energy to shift hunger and fullness ratings one point (*M* = 719 KJ) on Day 4, relative to the Control group (*M* = 427 KJ).

### Biological measures and their relationships

#### Blood glucose

**Fig 3:**
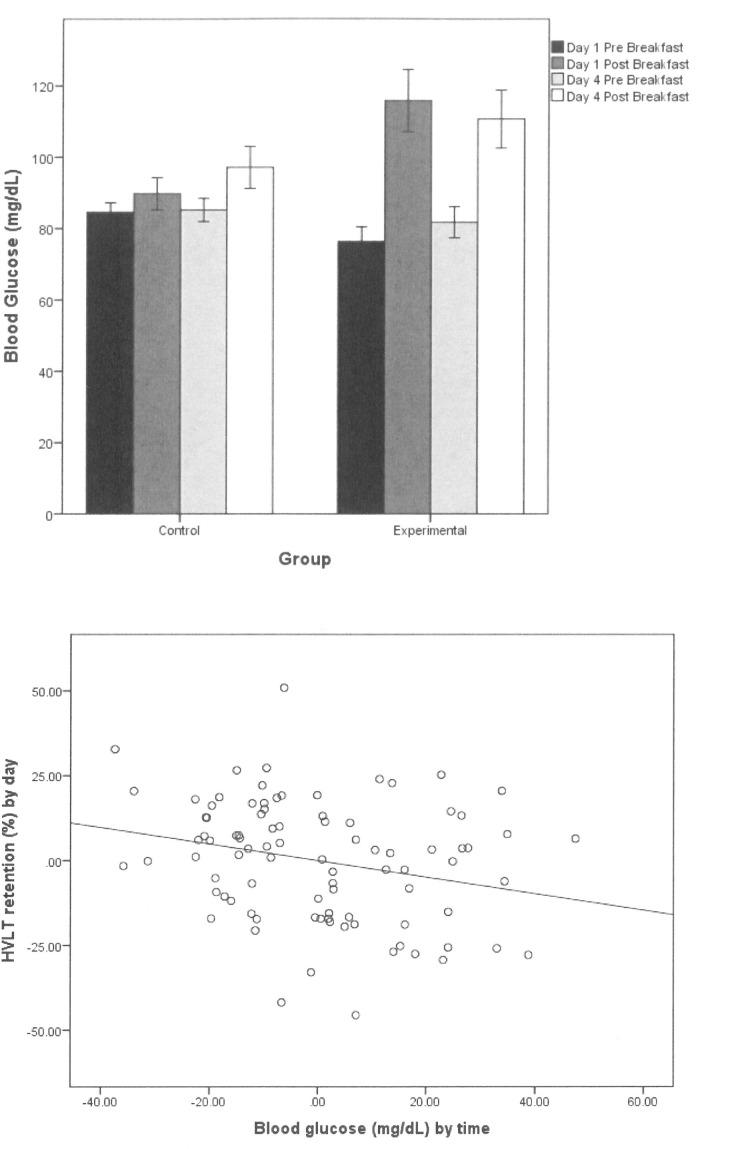
Blood glucose data. (A)—Mean (± standard error) pre- and post-prandial blood glucose levels at the start and end of the study for each group; (B)—Scatterplot of the negative linear relationship between HVLT retention score (%) by day (Day 4 –Day 1) and blood glucose levels (mg/dL) by time (post-breakfast–pre-breakfast).

Blood glucose data were analysed using the same three-way mixed design ANCOVA as described for the neuropsychological data. The ANCOVA revealed a main effect of Time (*F*(1,83) = 135.1, partial eta-squared = 0.62, *p* < 0.001), and an interaction of Time by Group (*F*(1,83) = 15.82, partial eta-squared = 0.16, *p* < 0.001). **Fig 3A** illustrates the Time by Group effect. It is evident that blood glucose readings increase across a meal (Time effect), and to a considerably greater extent in the Experimental group than for Controls (Time by Group).

We then examined whether the Group-related blood glucose effect (i.e., change across Time) was associated with changes in the neuropsychological measures of memory and the measure of interoception. To assess this, we used partial correlations, controlling for BMI, waist circumference, DFS score, gender, and either average breakfast energy intake (for the memory correlation) or Day 1 interoception score (for the interoception correlation).

A larger decline in HVLT score between Day 1 and Day 4 was significantly associated (Partial *r*(83) = -0.24, *p* = 0.028) with greater increase in blood glucose across the breakfasts (i.e., main effect of Time; see **Fig 3B**). There was no significant relationship between the interoception score and change in blood glucose. To determine whether changes in HVLT performance were mediated by blood glucose alterations, we also conducted a three-way ANCOVA, as with the neuropsychological measures, and included blood glucose change across time as an additional covariate. As expected, taking into account blood glucose change between meals, the Day by Group interaction for HVLT retention was not statistically significant, (*F*(1,82) = 3.11, partial eta-squared = 0.037, *p* = 0.082).

#### Triglycerides

**Fig 4:**
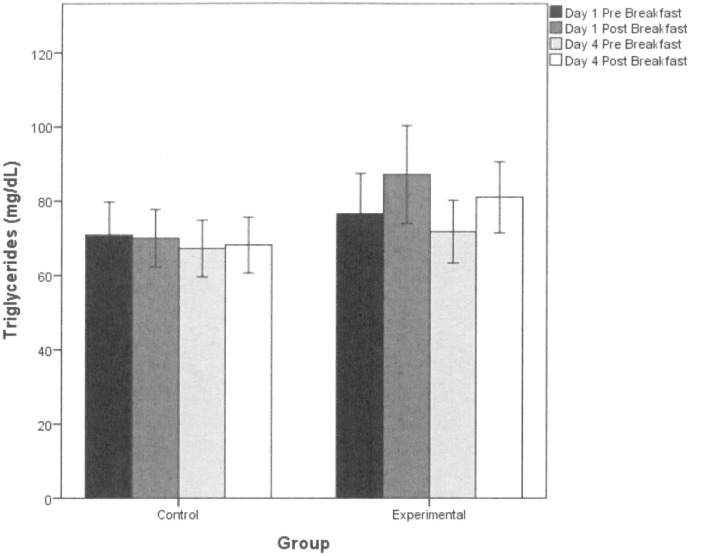
Triglycerides data. Mean (± standard error) pre- and post-prandial triglyceride levels at the start and end of the study for each group.

Similar to the blood glucose findings, the ANCOVA on the triglycerides data revealed main effects of Time (*F*(1,83) = 16.29, partial eta-squared = 0.16, *p* < 0.001) and Time by Group (*F*(1,83) = 7.23, partial eta-squared = 0.08, *p* = 0.009). As can be seen in **Fig 4**, blood triglycerides tended to increase after a meal (main effect of Time) and to a significantly greater extent in the Experimental group relative to the Controls (Time by Group). There were no significant partial correlations between changes in blood triglycerides across Time and neuropsychological or interoception measures.

#### Anthropometric data

There were no significant differences in BMI or waist circumference across the experiment, between groups. Changes in BMI and waist circumference were not significantly associated with the neuropsychological or interoception measures.

### Food diary data

The descriptive statistics for the nutrient profile of the food diaries (averaged across the two entries for the pre and during-study periods) are provided in **Table 4**. The dietary data were analysed using a three-way mixed design ANCOVA, with Week (Week 1[pre-study] vs. Week 2 [during study]) and Time (breakfast vs. post-breakfast) as the within factors and Group (Control vs. Experimental) as the between factor, with BMI, waist circumference, DFS score, and gender as covariates.

**Table 4:** Nutritional breakdown of self-report food diaries prior to and during the study.

	Descriptive statistics, Mean (SD)				Week by Group
	Pre-study		During study		
	Exp. (n = 48)	Control (n = 48)	Exp. (n = 48)	Control (n = 48)	p-value
Volume	2180.8 (1018.2)	2164.9 (927.9)	2117.5 (645.9)	2216.9 (853.9)	.04*
Energy (kJ)	7766.9 (1937.4)	7627.9 (2192.5)	8463.4 (1833.6)	8522 0 (2069.9)	.59
Total Fat (%)	35.0 (7.6)	35.0 (5.9)	42.0 (5.0)	27.7 (5.4)	.00*
Saturated fat (%)	13.1 (3.7)	13.1 (3.3)	19.5 (3.1)	9.8 (2.9)	.00*
Total carbohydrates (%)	43.7 (8.5)	42.1 (6.7)	40.6 (4.9)	39.9 (5.9)	.51
Sugars (%)	16.3 (6.2)	16.6 (6.3)	15.9 (4.9)	14.0 (4.7)	.36
Protein (%)	18.5 (4.7)	20.2 (5.8)	15.6 (3.4)	30.5 (6.8)	.00*

Exp. = Experimental group

Sig* = significance level p < .05 of repeated measures ANCOVA for Week (Week 1[pre-study] vs Week 2 [during study]) by Group interaction; kJ = kilojoules.

**Fig 5:**
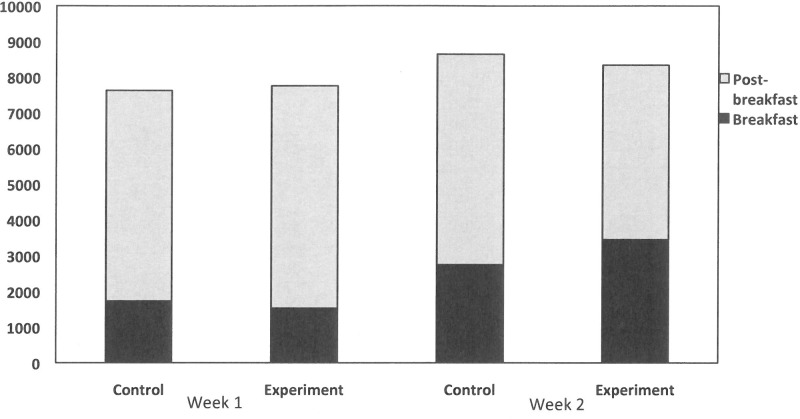
Food diary data. An illustration of the compensation of energy intake across weeks and between groups, following the breakfast manipulation. Overall energy intake is greater in Week 2 relative to Week 1, with no differences between groups (i.e., caloric compensation).

For energy intake, the ANCOVA revealed a main effect of Week (*F*(1,91) = 11.42, partial eta-squared = 0.11, *p* = 0.001), a main effect of Time (*F*(1,91) = 375.45, partial eta-squared = 0.81, *p* < 0.001), and a significant three-way interaction between Time, Week and Group, (*F*(1,90) = 18.08, partial eta-squared = 0.17, *p* < 0.001). Importantly, there was a non-significant Week by Group interaction, (*F*(1,90) = 0.29, partial eta-squared = 0.003, *p* = 0.59). Despite consuming more energy at breakfast (shown earlier), the Experimental group consumed significantly less energy the rest of the day (i.e., caloric compensation) during the experimental period, relative to the Control group (See **Fig 5**).

The Experimental group appear to have achieved this by reducing carbohydrate and sugar intake post-breakfast. Thus, while carbohydrate intake was significantly greater at breakfast in the Experimental group relative to the Control group, as with added sugar, overall intake did not differ between groups either before or during the experiment (see **Table 4**).

The Experimental group did not show compensation over the course of the day for total fat, saturated fat, or protein intake (see **Table 4)**. That is, relative to intake prior to the study, there were significant increases in the Experimental group in total fat, (*F*(1,90) = 21.04, partial eta-squared = 0.19, *p* < 0.001), saturated fat, (*F*(1,90) = 60.27, partial eta-squared = 0.40, *p* < 0.001), and in protein, (*F*(1,90) = 73.59, partial eta-squared = 0.45, *p* < 0.001) relative to the Control group.

In sum, while energy, fat and sugar intakes were significantly higher at breakfast in the Experimental group providing a discrete ‘burst’ of these nutrients, group differences in energy and sugar intake were not evident when the whole experimental days intake was computed. Across the whole of the experimental days, only differences in fat and saturated fat and protein were evident.

## Discussion

The primary aim of this study was to determine if an HFS diet causes poorer performance on tests of HDLM. Additionally, we examined for changes in interoceptive sensitivity for hunger and fullness, as well as testing whether any observed changes in behavioural measures were associated with biological markers (i.e., blood glucose and lipid measures). Our key findings were: (1) a brief four-day HFS intervention led to significantly poorer memory retention, relative to the Control group, on the HVLT test, but not on the LM test; (2) the magnitude of this change in HVLT performance was significantly associated with the change in blood glucose across the experimental meals; (3) the Experimental group became significantly less sensitive to the effects of the breakfast, requiring more energy to shift hunger and fullness ratings an equivalent amount on Day 4 relative to Day 1; and (4) the Experimental group did not consume more energy than the Controls overall, despite consuming significantly more energy at breakfast. Most of this compensation was for carbohydrate intake, as the Experimental group still consumed more saturated fat.

The principal finding from this study was the decline in performance on the HVLT retention score in the Experimental group, relative to the Control group. This is the first experimental evidence in humans that a brief dietary manipulation of saturated fat and added sugar intake leads to poorer performance on tests known to be hippocampally-related. To the best of our knowledge, this is the first experimental study in humans to parallel the findings in the animal literature. Moreover, the reduction in HDLM performance (as in the animal data) was evident after a relatively brief period of exposure to the diet. Another important element of this finding is that HDLM impairments were independent of energy intake, and thus were a consequence of the macronutrient profile of the diet. Again, similar to animals, impairments in HDLM occurred in a healthy and lean population. Importantly, performance on non-hippocampal control measures did not deteriorate as a function of diet (noting that this is based on a limited set of such measures–forward and backward digit span), suggesting that HFS diets impact hippocampal measures specifically. Indeed, this diet-related specificity would likely be supported further by the use of multiple non-hippocampal measures in future studies.

The food diary data indicate the Experimental group, despite consuming more energy at breakfast than the Control group, did not consume more energy overall relative to the Control group. While daily energy intake was comparable, macronutrient intake differed between groups. Specifically, there were no difference in *daily* carbohydrate and sugar intake (i.e., evidence of compensation), while *daily* total and saturated fat intake remained elevated. An interesting question raised by the food diary data is what particular aspect of the dietary manipulation led to the change in HVLT performance? There are at least three possibilities.

The first is that the breakfast ‘burst’ of saturated fat and added sugar in the Experimental group was the causative factor. Indeed, tests of verbal memory–known to be hippocampally-based–appear to be most sensitive to experimental manipulations of macronutrient content [37, 46]. Likewise, the design of this study (i.e., a brief exposure to an HFS diet giving a ‘burst’ of saturated fats and refined sugars) is consistent with rodent studies showing that animals given restricted access to HFS foods show similar deficits in learning and memory performance (e.g., [81, 82]). The second possibility is that the greater net intake of saturated fat and total fat inside and outside of the laboratory in the Experimental group–across all four experimental days–represents the causative factor. The third possibility is that overall saturated fat and added sugar intake (and perhaps overall energy intake) were *actually* higher in the Experimental group, but this was disguised deliberately or otherwise by dietary underreporting. We would suggest that the first alternative may be the most plausible, simply because we observed a relationship between changes in blood glucose across breakfast and change in HVLT performance across Days, with additional evidence that changes in blood glucose mediated alterations in HVLT performance. This would suggest that it was something about the breakfasts that led to changes in HDLM. The second possibility cannot, however, be discounted. Diets rich in just saturated fat can lead to impairments in HDLM in animals [11–14] and are associated with poorer memory recall in young women [10], and so this could represent either an alternative or additional route to changes in HVLT performance in the Experimental group. However, given that few human studies have effectively manipulated saturated fat intake [37], it is unclear whether saturated fat or sugar intake alone evoked such changed in HDLM performance. While the third account is plausible it may be the least likely. Dietary underreporting seems to be a more persistent phenomena for the obese [83], with healthy weight participants generally being more accurate reporters. Finally, while we note that protein intake also differed between the Groups, we are not aware of any mechanism by which this macronutrient could generate the observed outcomes.

An unexpected finding was that while the HVLT was sensitive to changes in dietary fat and sugar intake, the LM measure showed no such change. One possible reason for this difference may arise in the appropriateness of using alternate forms of each test. The HVLT has six alternate forms that make it ideal for repeat testing, and these have been cross-validated and are indeed recommended and considered appropriate to be used in such a manner. On the other hand, the alternate forms of the LM test were taken from [73] and [74], which have been validated against the LM test from the Wechsler Memory Scale 4^th^ edition and the Wechsler Memory Scale 3^rd^ edition, respectively. Importantly, while multiple forms of the LM test were required for this study, these alternate forms have not been validated against each other, making the assumption of equivalency across these alternate forms *potentially* problematic. That alternate forms may be a problem for the LM test comes from consideration of coefficient alpha. For the HVLT this was adequate (α = 0.70), but it was poor for LM retention (α = 0.52). It may ultimately be this poorer reliability that accounts for the failure to detect any diet-related changes with LM.

The Experimental group breakfasts led to far greater increases in blood glucose and triglycerides than the breakfasts consumed by the Control group. Furthermore, controlling for meal-related changes in these markers removed the difference between groups in HVLT performance across days, suggesting that significant alterations in these markers may contribute to changes in cognitive performance. That blood glucose and triglycerides show a similar pattern of change is not surprising. Glucoregulation is associated with lipid regulation [84], and consumption of HFS foods leads to marked increases in both blood glucose and triglyceride levels [85,86]. As we noted in the Introduction, impaired glucoregulation may represent one putative causal pathway by which an HFS diet may adversely affect the hippocampus. Consistent with this possibility, we observed a significant association between blood glucose changes across the test breakfasts and changes in HDLM on the HVLT across the course of the study. Whether this effect is mediated directly by changes in blood glucose or indirectly via some other mechanism such as inflammation, remains to be established. Support for an inflammatory mediation account comes from the well-established relationship between inflammation and impaired HDLM [87,88]. In addition, animal studies show that diet-induced impairments in HLDM are linked to increased levels of inflammation [2,18,55,56,89] and changes in blood glucose concentration are correlated with elevated markers of inflammation in the hippocampus, but not the perirhinal cortex or hypothalamus [18]. Likewise, a diet high in processed meat, refined grains and sugar-sweetened beverages is strongly related to inflammatory markers in women with type-2 diabetes [90], and increasing sucrose intake in overweight individuals increases inflammation [91]. Recent evidence suggests that poor diet may also contribute to neuroinflammation and neurodegeneration (see [92]). The potential link between glucoregulation and inflammation may be a causative factor in diet-induced impairments in HDLM, but remains to be experimentally verified for the paradigm used here.

Another consequence of shifting healthy subjects to an HFS diet was that the Experimental group showed reduced sensitivity over days to changes in hunger and fullness following breakfast consumption. The Experimental group required more energy on Day 4 than on Day 1 to shift hunger and fullness ratings an equivalent amount relative to the Control group. Our findings parallel correlational research in humans linking HFS diet and an impaired sensitivity to hunger and fullness [7–9]. What this suggests is that an HFS diet not only impairs performance on hippocampal-related memory tasks, but also the ability to accurately sense changes in hunger and fullness. It has been argued that the regulation of appetitive behaviour is based on the ability of satiety to inhibit food-related associations and this ability depends on the functional integrity of the hippocampus [3]. A Western-style diet impacts hippocampal function and therefore the ability of satiety cues to inhibit this association, ultimately leading to excess weight gain [3]. Here, we provide evidence of impaired sensitivity to hunger and fullness following experimental manipulation of HFS intake in healthy lean humans.

A further issue and one not addressed in the current study, or indeed in the wider animal literature, is the reversibility of the changes in HDLM. That these changes are reversible seems likely. This is because several factors are known to up-regulate neurogenesis and relatedly hippocampal-related functioning in animals and these include exercise, environmental enrichment and ant-depressant medications [93,94]. At least in the case of anti-depressants, these may exert some of their therapeutic effect by returning the hippocampus to a pre-depression state of functioning [93,94]. As the time course for anti-depressant effects to up-regulate neurogenesis and hippocampal functioning is between 4–6 weeks in humans and rodents [93,94], we would tentatively suggest that the recovery period from a dietary intervention such as the one used here *may* have a similar time course.

### Limitations

We are aware that there are at least three main limitations in conducting this study. First, one HDLM measure (i.e., Logical Memory) did not show diet-related changes, which we argue is related to appropriateness of its alternate forms. Furthermore, the specificity of this diet-related change to HDLM was supported by a limited number of non-hippocampal measures. Future research would benefit from the use of multiple, appropriate hippocampal and non-hippocampal measures. Second, self-report of dietary habits was used in the recruitment of participants and in the food dairy collection. One potential criticism is that self-report dietary measures may be problematic due to underreporting. This is based on the finding that, in comparing what one would need to eat to maintain current body weight and what people self-report, individuals typically underreport the amount of food they consume (e.g., [95]). Underreporting is also more common in individuals with a higher BMI (e.g., [96,97]), where factors such as social desirability may play a role. We addressed this potential concern by using a lean healthy-eating sample population who would likely be more accurate in their recall (e.g., [9]) and less likely to underreport (e.g., [98]). Third, a lean healthy university population is likely more homogenous than the general population and this may limit external validity. However, we argue that a more heterogeneous sample may vary considerably on many factors that are also known to impact hippocampal functioning (e.g., body weight, dietary habits, physical activity, smoking etc.). A more homogenous sample, therefore, allows for greater control over these factors, thereby maximising the opportunity to observe diet-related changes in hippocampal-related functioning.

In conclusion, we show that brief consumption of a Western-style diet leads to impairments in HDLM and interoception in healthy lean young adults. Further, these changes in HDLM were linked to shifts in blood glucose across breakfast, suggesting one potential mechanism by which a Western-style diet can affect hippocampal function.

## Supporting information

S1 Data A CSV file containing all of the reported study data. (CSV)
